# Substance use and incidence of metabolic syndrome before midlife among military adults: the CHIEF cohort study

**DOI:** 10.3389/fpubh.2024.1406524

**Published:** 2024-06-04

**Authors:** Wei-Nung Liu, Yi-Chiung Hsu, Yen-Po Lin, Kun-Zhe Tsai, Yun-Chen Chang, Pang-Yen Liu, Gen-Min Lin

**Affiliations:** ^1^Department of Medicine, Hualien Armed Forces General Hospital, Hualien City, Taiwan; ^2^Department of Biomedical Sciences and Engineering, National Central University, Taoyuan, Taiwan; ^3^Department of Medicine, Tri-Service General Hospital, National Defense Medical Center, Taipei, Taiwan; ^4^Department of Stomatology of Periodontology, Mackay Memorial Hospital, Taipei, Taiwan; ^5^Department of Dentistry, Tri-Service General Hospital, National Defense Medical Center, Taipei, Taiwan; ^6^School of Nursing and Graduate, Institute of Nursing, China Medical University, Taichung, Taiwan; ^7^Department of Nursing, China Medical University Hospital, Taichung, Taiwan

**Keywords:** cohort study, metabolic syndrome, substance use, military personnel, physical activity

## Abstract

**Backgrounds:**

Habitual substance use, i. e., alcohol, tobacco and betel nut, has been found with an increased risk of metabolic syndrome (MetS) in the general population, whereas the association remains unclear in physically fit military personnel. This study aimed to investigate the combination of these substances use and their associations with new-onset MetS in the military.

**Methods:**

A total of 2,890 military men and women, aged 18–39 years, without MetS were obtained from the cardiorespiratory fitness and health in eastern armed forces study (CHIEF) in Taiwan and followed for incident MetS from baseline (2014) through the end of 2020. Incident MetS event was defined by the International Diabetes Federation guideline and confirmed in the annual health examinations. A self-report was used to assess the alcohol, tobacco and betel nut use status (active vs. former/never). Multivariable Cox regression model was performed to determine the association with adjustments for sex, age, body mass index and physical activity at baseline.

**Results:**

At baseline, there were 279 active betel nut chewers (9.7%), 991 active smokers (34.3%) and 1,159 active alcohol consumers (40.1%). During a mean follow-up of 6.0 years, 673 incident MetS (23.3%) were observed. As compared to no substance users, only one substance, and two and three substances users had a greater risk of incident MetS [hazard ratios (HRs) and 95% confidence intervals: 1.27 (1.06–1.54), 1.38 (1.12–1.69) and 1.78 (1.37–2.32), respectively]. In subgroup analyses, the risk of incident MetS in two and three substances users was significantly greater in those free of baseline low high-density lipoprotein [HRs: 1.54 (1.21–1.95) and 2.57 (1.92–3.46), respectively], as compared to their counterparts (both p for interactions <0.05).

**Conclusion:**

A dose-response association of more substances use for new-onset MetS was noted in military personnel. This finding suggests that the combined alcohol, tobacco and betel nut use may play a role in the development of MetS. Further study is required to establish causation and to investigate the potential benefits of substance use cessation in reducing the risk of MetS.

## Introduction

Metabolic syndrome (MetS) is a cluster of interrelated risk factors that increase an individual's susceptibility to developing cardiovascular disease and type 2 diabetes mellitus (1–3). The prevalence of MetS has been on the rise globally, and it has become a significant public health concern (4, 5). Numerous studies have investigated the association between various lifestyle factors, such as alcohol consumption, smoking, and betel nut chewing, and the development of MetS (6–10). However, the synergistic effect of these unhealthy lifestyle behaviors on the incidence of MetS, particularly among physically active young adults, has not been thoroughly explored.

Prior research has shown that excessive alcohol consumption can contribute to the development of MetS by increasing the risk of obesity, insulin resistance, and dyslipidemia (1, 11–16). Similarly, cigarette smoking has been linked to a higher prevalence of MetS, possibly related to its effects on lipid metabolism, insulin sensitivity, and abdominal obesity (3, 11, 17–19). Betel nut chewing, a common practice in many Asian countries, has also been associated with an increased risk of MetS, as it may lead to obesity, insulin resistance, and impaired glucose tolerance (6, 7, 9, 20–23).

While the individual effects of these unhealthy lifestyle behaviors on MetS have been studied, there is limited research on the synergistic impact of these factors, especially in military populations who were characterized by physically fit and prevalent with substances use. Understanding the synergistic effect of multiple unhealthy lifestyle behaviors on the incidence of MetS is crucial for developing targeted preventive strategies and interventions. Accordingly, this study aimed to investigate the association of the combined use of alcohol, tobacco, and betel nut with the incidence of MetS among military men and women in Taiwan. By examining the combined effect of these unhealthy lifestyle behaviors, we hope to provide valuable insights into the development of MetS and inform future preventive measures targeting military young adults.

## Methods

### Study population

This study utilized data from the cardiorespiratory fitness and health in eastern armed forces (CHIEF) study, a prospective longitudinal cohort study aimed to clarify the association between physical fitness and cardiometabolic health in military personnel in Taiwan (9, 24–28). This study consists of 4,080 participants of 18–50 years old, recruited from various military bases in Hualien and Taitung Counties in Taiwan, and underwent annual health examinations between 2014 and 2020. The study protocol has been approved by the Institutional Review Board of the Mennonite Christian Hospital (certificate no. 16-05-008) in Taiwan. Written informed consent was obtained from all participants.

### Clinical and demographic measures

Anthropometric measurements, including body weight (kg), height (m), and waist circumference (cm), were objectively obtained with participants standing erect, abdomen relaxed, arms at their sides, and feet together. Body mass index (BMI) was calculated as weight in kilograms divided by height in meters squared (kg/m^2^). Hemodynamic variables of systolic blood pressure (BP) and diastolic BP, were measured once after a rest for 15 min or more over the right arm with participants seated, utilizing a standardized automatic device (Parama-Tech Co., Ltd, Fukuoka, Japan). If the BP was ≥130 mmHg in systolic and ≥80 mmHg in diastolic, a second measurement of BP would be performed following a rest for 15 min, and an average of sum of the two BP levels was treated as the final BP level. Following a 12-h overnight fast, peripheral venous blood samples were collected from the antecubital fossa. Biochemical parameters, i.e., total cholesterol, low-density lipoprotein cholesterol (LDL-C), high-density lipoprotein cholesterol (HDL-C), triglycerides, fasting glucose (FPG), and uric acid, were assayed using an Olympus AU640 autoanalyzer (Olympus, Kobe, Japan) (9, 25–28).

### Definition of substance use status

Substance use was assessed using a self-administered questionnaire at baseline. The betel-nut chewing, cigarette smoking and alcohol consumption status were categorized as current active and former or never in the past 6 months. Active tobacco use was defined as smoking at least 20 cigarettes per week. Active betel nut chewing was defined as consuming betel nut at least once per week. Since alcohol consumption was forbidden on military bases in Taiwan, active alcohol consumption was defined as drinking at least once per day during holidays or vacations. Former users were defined as ever active substance users before 6 months and cessation of the substance for 6 months.

### Definition of incident MetS

Incident MetS was identified based on the International Diabetes Federation (IDF) guidelines (29), which require the presence of abdominal obesity (waist circumference ≥90 cm for men and ≥80 cm for women) plus any two of the following four features: (1) elevated triglycerides (≥150 mg/dL) or specific treatment for this lipid abnormality; (2) reduced HDL-C (<40 mg/dL for men and <50 mg/dL for women) or specific treatment for this lipid abnormality; (3) elevated BP (systolic ≥130 mmHg and/or diastolic ≥85 mmHg) or treatment of previously diagnosed hypertension; and (4) elevated FPG (≥100 mg/dL) or previously diagnosed type 2 diabetes. MetS events were ascertained through annual health examinations and medical records during the follow-up period (2014–2020).

### Statistical analysis

Participants were categorized into four groups based on their substance use: non-substance users, single substance users, two-substance users (a combination of two substances), and three-substance users (alcohol, tobacco, and betel nut). Baseline characteristics were compared among the substance use groups using two-way ANOVA for continuous variables and chi-square tests for categorical variables. The Cox proportional hazards regression model was used to estimate the hazard ratios (HRs) and 95% confidence intervals (CI) for the association of substance use status and risk of MetS. Multivariable model 1 was adjusted for potential covariates of age, sex and physical activity level. Multivariable model 2 was adjusted for the covariates in model 1 plus BMI. Multivariable model 3 was adjusted for the covariates in model 1 plus waist circumference. Subgroup analyses were stratified by baseline metabolic features (BP, waist circumference, HDL-C, serum triglycerides, and FPG) to explore potential effect modification. The Kaplan-Meier curve was used to assess the event-free survival probabilities for the different substance use groups and the combined substances groups. All statistical analyses were performed using SPSS software v25.0 for Windows (IBM Corp., Armonk, NY, USA).

## Results

In this cohort study, participants who had established MetS (*N* = 457), those lost to follow-up due to transfer out of military bases in Eastern Taiwan (*N* = 675) and those aged 40 years or older at baseline (*N* = 58) were excluded, leaving a final sample of 2,890 male and female participants for the present analysis.

The baseline characteristics of the study participants, stratified by the substance use status, are presented in Table 1. Of the 2,890 participants, 1,366 (47.3%) reported no substance use, 815 (28.2%) reported single substance use, 511 (17.7%) reported two-substance use, and 198 (6.9%) reported three-substance use. Significant differences were observed among the groups in terms of age, sex, physical activity level, BMI, waist circumference, BP, HDL-C, triglyceride, and FPG. Notably, there were a greater proportion of women and those with physical activity <150 min/week. In addition, the mean levels of BMI, waist circumference, BP, lipid profile and FPG were lower in no substance users.

**Table 1:** Baseline characteristics of study participants by the combined substances use status (*N* = 2,890).

	**No substance use (*N =* 1,366)**	**Combined substance use status (*N* = 1,524)**			***p***
		**One substance use (*****N** =* **815)**	**Two substances use (*****N** =* **511)**	**Three substances use (*****N** =* **198)**	
Alcohol drinking, %	0 (0.0)	487 (59.8)	476 (93.2)	198 (100)	<0.001
Betel-nut chewing, %	0 (0.0)	10 (1.2)	71 (13.9)	198 (100)	<0.001
Cigarette smoking, %	0 (0.0)	318 (39.0)	475 (93.0)	198 (100)	<0.001
Age, years	27.77 ± 5.89	29.05 ± 5.52	29.05 ± 5.81	28.09 ± 5.75	<0.001
**Sex, %**					
Men	1,133 (82.9)	756 (92.8)	496 (97.1)	196 (99.0)	<0.001
Women	233 (17.1)	59 (7.2)	15 (2.9)	2 (1.0)	
**PA level, %**					
<150 min/week	324 (23.7)	133 (16.3)	119 (23.3)	51 (25.8)	<0.001
150–299 min/week	481 (35.2)	357 (43.8)	197 (38.6)	69 (34.8)	
≥300 min/week	561 (41.1)	325 (39.9)	195 (38.2)	78 (39.4)	
BMI, kg/m^2^	24.05 ± 3.04	24.35 ± 2.86	24.47 ± 2.98	24.95 ± 3.14	<0.001
Waist circumference, cm	80.34 ± 8.28	82.08 ± 7.37	82.46 ± 8.22	83.63 ± 7.79	<0.001
Systolic BP, mmHg	115.10 ± 13.08	116.91 ± 12.68	116.08 ± 13.09	116.67 ± 11.72	0.01
Diastolic BP, mmHg	68.73 ± 9.34	70.24 ± 9.88	69.21 ± 10.00	69.53 ± 9.55	0.006
**Blood tests**					
Total cholesterol, mg/dL	170.73 ± 30.82	173.29 ± 33.09	172.20 ± 35.25	174.69 ± 34.62	0.19
LDL-C, mg/dL	102.46 ± 27.40	105.36 ± 29.70	104.16 ± 31.10	106.08 ± 30.30	0.08
HDL-C, mg/dL	50.85 ± 10.35	49.67 ± 9.90	48.79 ± 9.21	47.57 ± 9.32	<0.001
Triglyceride, mg/dL	89.86 ± 53.55	96.27 ± 52.61	106.07 ± 64.72	124.81 ± 84.15	<0.001
FPG, mg/dL	91.34 ± 8.53	92.31 ± 11.26	92.50 ± 9.06	93.87 ± 11.86	0.001
**Metabolic abnormality**					
Blood pressure ≥130/85 mmHg, %	205 (15.0)	136 (16.7)	85 (16.6)	30 (15.2)	0.69
Elevated waist size, % ≥90 cm in men; ≥80 cm in women	235 (17.2)	138 (16.9)	93 (18.2)	36 (18.2)	0.92
Declined HDL-C, % <40 mg/dL in men; <50 mg/dL in women	213 (15.6)	116 (14.2)	72 (14.1)	32 (16.2)	0.73
TG ≥150 mg/dL, %	126 (9.2)	85 (10.4)	75 (14.7)	51 (25.8)	<0.001
FPG ≥100 mg/dL, %	150 (11.0)	105 (12.9)	70 (13.7)	35 (17.7)	0.03

Continuous variables are expressed as mean ± standard deviation (SD), and categorical variables as N (%).

PA, physical activity; BMI, body mass index; BP, blood pressure; LDL-C, low-density lipoprotein cholesterol; HDL-C, high-density lipoprotein cholesterol; TG, triglyceride; FPG, fasting plasma glucose.

During a mean follow-up of 6.0 years, 673 incident MetS cases (23.3%) were found. The multivariable Cox regression analysis results for the incidence of MetS with combined substance use are shown in Table 2. Compared with non-substance users, single substance users had a significantly greater risk of new-onset MetS in the crude model and multivariable Models 1–3 [HRs and 95% CIs: 1.44 (1.20–1.73), 1.24 (1.03–1.49), 1.27 (1.06–1.54), and 1.21 (1.00–1.46), respectively]. In addition, two-substance users had a significantly greater risk of new-onset MetS in the crude model and multivariable Models 1–3 [HRs: 1.64 (1.33–2.01), 1.37 (1.11–1.68), 1.38 (1.12–1.69), and 1.28 (1.04–1.58), respectively]. Moreover, three-substance users had a greater risk of new-onset MetS in the crude model and multivariable Models 1–3 [HRs: 2.15 (1.65–2.80), 1.93 (1.48–2.52), 1.78 (1.37–2.32), and 1.78 (1.37–2.33), respectively]. Notably, there were graded higher associations for incident MetS with a combination of more substance use in all models (all p-values for trend <0.001).

**Table 2:** Multivariable cox regression analysis for incidence of metabolic syndrome with combined substances use.

	**N**	**Events**	**Crude model**			**Multivariable model 1**			**Multivariable model 2**			**Multivariable model 3**		
			**HR**	**95% CI**	***p***	**HR**	**95% CI**	***p***	**HR**	**95% CI**	***p***	**HR**	**95% CI**	***p***
No substance use	1,366	242	1.00			1.00			1.00			1.00		
One substance use	815	211	1.44	1.20–1.73	<0.001	1.24	1.03–1.49	0.02	1.27	1.06–1.54	0.01	1.21	1.00– 1.46	0.04
Two substances use	511	148	1.64	1.33–2.01	<0.001	1.37	1.11–1.68	0.003	1.38	1.12–1.69	0.003	1.28	1.04–1.58	0.01
Three substances use	1,98	72	2.15	1.65–2.80	<0.001	1.93	1.48–2.52	<0.001	1.78	1.37–2.32	<0.001	1.78	1.37–2.33	<0.001
p-value for trend					<0.001			<0.001			<0.001			<0.001

Data are presented as hazard ratio (HR) and 95% confidence interval (CI).

Multivariable Model 1 was adjusted with age, sex and physical activity level.

Multivariable Model 2 was adjusted with age, sex, physical activity level and body mass index.

Multivariable Model 3 was adjusted with age, sex, physical activity level and waist circumference.

The multivariable Cox regression analysis results for the incidence of MetS with specific substance use are presented in Table 3. Among the single substance users, there were 487 active alcohol consumers, 10 active betel nut chewers, and 318 active smokers. Active alcohol consumption was associated with a greater risk of incident MetS in crude model and in multivariable models 1 and 2 [HRs: 1.49 (1.20–1.84), 1.27 (1.02–1.57), and 1.24 (1.00–1.55), respectively], whereas the strength of the association was modestly attenuated in Model 3 [HR: 1.21 (0.97–1.50), *p* = 0.08]. Since there were only 10 participants who exclusively chewed betel nut, all the positive associations in crude model and Model 1 [HRs: 1.45 (0.47–4.53) and 1.13 (0.36–3.53), respectively] and the inverse associations in Model 2 and Model 3 [HRs: 0.58 (0.18–1.82) and 0.60 (0.19–1.90), respectively] were not statistically significant. Active cigarette smoking was associated with a greater risk of new-onset MetS in crude model and in multivariable models 2 and 3 [HRs: 1.37 (1.06–1.77), 1.46 (1.13–1.89), and 1.33 (1.02–1.72), respectively].

**Table 3:** Multivariable cox regression analysis for incidence of metabolic syndrome with specific substance use.

	**N**	**Events**	**Crude Model**			**Model 1**			**Model 2**			**Model 3**		
			**HR**	**95% CI**	***p***	**HR**	**95% CI**	***p***	**HR**	**95% CI**	***p***	**HR**	**95% CI**	***p***
No substance use	1,366	242	1.00			1.000			1.00			1.00		
Only alcohol drinking	487	131	1.49	1.20–1.84	<0.001	1.27	1.02–1.57	0.03	1.24	1.00–1.54	0.04	1.21	0.97–1.50	0.08
Only betel nut chewing	10	3	1.45	0.47–4.53	0.52	1.13	0.36–3.53	0.83	0.58	0.18–1.82	0.35	0.60	0.19– 1.90	0.38
Only cigarette smoking	318	77	1.37	1.06–1.77	0.01	1.25	0.97–1.62	0.09	1.46	1.13–1.89	0.004	1.33	1.02–1.72	0.03

Data are presented as hazard ratio (HR) and 95% confidence interval (CI).

Multivariable Model 1 was adjusted with age, sex and physical activity level.

Multivariable Model 2 was adjusted with age, sex, physical activity level and body mass index.

Multivariable Model 3 was adjusted with age, sex, physical activity level and waist circumference.

Table 4 presents the results of the subgroup analyses, stratified by various metabolic features at baseline. The associations did not differ in most subgroup analyses, except that the associations of two- and three-substance use with the risk of MetS were significantly greater in those without baseline low HDL-C in multivariable model 2 [HRs: 1.54 (1.21–1.95) and 2.57 (1.92–3.46), respectively] compared to those with baseline low HDL-C (both p for interaction <0.05).

**Table 4:** Multiple cox regression analysis for incidence of metabolic syndrome with unhealthy lifestyle behaviors.

	**Multivariable model 1**							**Multivariable model 2**						
	**HR**	**95% CI**	**p**	**HR**	**95% CI**	**p**	**p-value for interaction**	**HR**	**95% CI**	**p**	**HR**	**95% CI**	**p**	**p-value for interaction**
**Baseline BP**<**130/85 mmHg (*****N** =* **2,434)**			**Baseline BP** ≥**130/85 mmHg (*****N** =* **456)**				**Baseline BP**<**130/85 mmHg (*****N** =* **2,434)**			**Baseline BP** ≥**130/85 mmHg (*****N** =* **456)**			
One substance use	1.46	1.18–1.81	<0.001	1.30	0.90–1.88	0.17	0.63	1.27	1.02–1.57	0.03	1.17	0.81–1.71	0.40	0.74
Two substances use	1.77	1.40–2.23	<0.001	1.21	0.78–1.87	0.39	0.13	1.44	1.14–1.83	0.002	1.23	0.79–1.91	0.36	0.49
Three substances use	2.42	1.81–3.23	<0.001	1.34	0.70–2.54	0.37	0.11	2.08	1.55–2.79	<0.001	1.57	0.82–3.01	0.17	0.44
	**Baseline waist girdle**<**90 cm in men;**<**80cm in women (*****N** =* **2,388)**			**Baseline waist girdle** ≥**90 cm in men;** ≥**80 cm in women (*****N** =* **502)**				**Baseline waist girdle**<**90 cm in men;**<**80 cm in women (*****N** =* **2,388)**			**Baseline waist girdle** ≥**90 cm in men;** ≥**80 cm in women (*****N** =* **502)**			
One substance use	1.56	1.24–1.96	<0.001	1.28	0.93–1.75	0.12	0.31	1.35	1.07–1.70	0.01	1.11	0.81–1.53	0.51	0.26
Two substances use	1.73	1.34–2.23	<0.001	1.49	1.06–2.10	0.02	0.52	1.44	1.11–1.87	0.005	1.23	0.87–1.75	0.24	0.37
Three substances use	2.30	1.67–3.17	<0.001	2.27	1.42–3.63	0.001	0.87	2.05	1.49–2.83	<0.001	1.92	1.19–3.09	0.008	0.83
	**Baseline HDL-C** ≥**40 mg/dL in men;** ≥**50 mg/dL in women (*****N** =* **2,457)**			**Baseline HDL-C**<**40 mg/dL in men;**<**50 mg/dL in women (*****N** =* **433)**				**Baseline HDL-C** ≥**40 mg/dL in men;** ≥**50 mg/dL in women (*****N** =* **2,457)**			**Baseline HDL-C**<**40 mg/dL in men;**<**50 mg/dL in women (*****N** =* **433)**			
One substance use	1.61	1.30–2.00	<0.001	1.13	0.79–1.63	0.50	0.11	1.36	1.09–1.69	0.006	1.00	0.67–1.45	0.98	0.12
Two substances use	1.85	1.46–2.35	<0.001	1.18	0.78–1.76	0.43	0.06	1.54	1.21–1.95	<0.001	0.91	0.60–1.38	0.65	0.01
Three substances use	2.81	2.10–3.77	<0.001	0.76	0.40–1.44	0.40	<0.001	2.57	1.92–3.46	<0.001	0.57	0.30–1.08	0.08	<0.001
	**Baseline TG**<**150 mg/dL (*****N** =* **2,553)**			**Baseline TG** ≥**150 mg/dL (*****N** =* **337)**				**Baseline TG**<**150 mg/dL (*****N** =* **2,553)**			**Baseline TG** ≥**150 mg/dL (*****N** =* **337)**			
One substance use	1.44	1.17–1.78	0.001	1.29	0.86–1.92	0.21	0.60	1.25	1.01–1.55	0.03	1.24	0.83–1.87	0.29	0.98
Two substances use	1.55	1.22–1.97	<0.001	1.35	0.90–2.03	0.14	0.56	1.33	1.04–1.69	0.02	1.28	0.85–1.94	0.24	0.77
Three substances use	2.05	1.48–2.84	<0.001	1.22	0.77–1.95	0.39	0.07	1.89	1.35–2.59	<0.001	1.24	0.77–2.00	0.37	0.23
	**Baseline FPG**<**100 mg/dL (*****N** =* **2,530)**			**Baseline FPG** ≥**100 mg/dL (*****N** =* **360)**				**Baseline FPG**<**100 mg/dL (*****N** =* **2,530)**			**Baseline FPG** ≥**100 mg/dL (*****N** =* **360)**			
One substance use	1.37	1.11–1.68	0.003	1.73	1.13–2.65	0.01	0.34	1.17	0.95–1.45	0.13	1.60	1.04–2.46	0.03	0.25
Two substances use	1.63	1.30–2.04	<0.001	1.55	0.96–2.51	0.07	0.89	1.35	1.08–1.70	0.01	1.46	0.89–2.39	0.13	0.98
Three substances use	2.10	1.56–2.83	<0.001	2.02	1.14–3.56	0.01	0.89	1.88	1.40–2.54	<0.001	1.91	1.08–3.39	0.02	0.98

Data are presented as hazard ratio (HR) and 95% confidence interval (CI).

Multivariable Model 1 was adjusted with age, sex and physical activity level.

Multivariable Model 2 was adjusted with age, sex, physical activity level and body mass index.

BP, blood pressure; HDL-C, high-density lipoprotein cholesterol; TG, triglycerides; FPG, fasting glucose.

**Figure 1:**
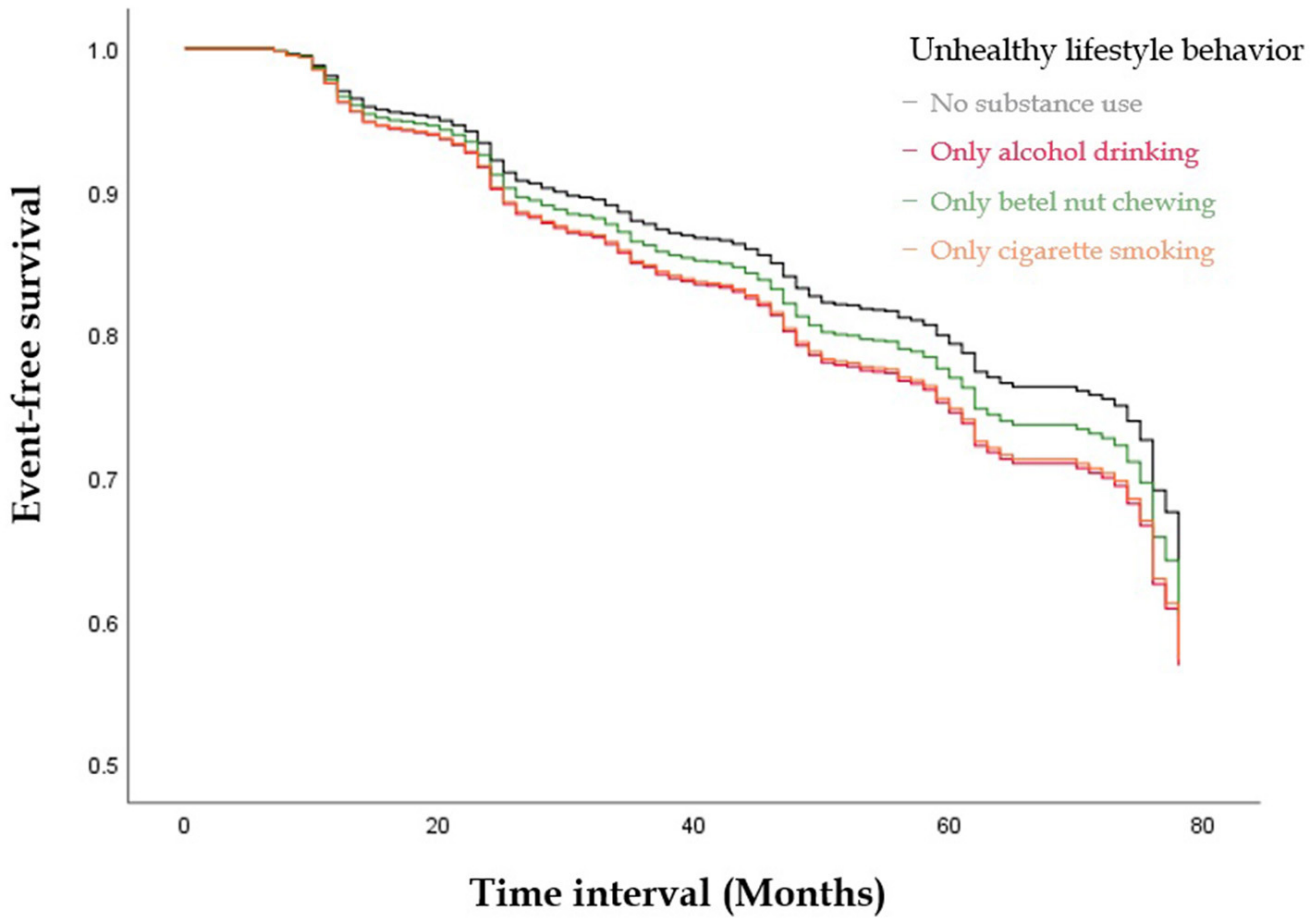
The Kaplan-Meier Curve illustrate the event-free survival curves for different substances use groups during the study period. Those who reported only active smoking and those who reported only alcohol consumption had the lowest event-free survival probability, followed by those who reported only active betel nut chewing as compared to those with no substance use.

**Figure 2:**
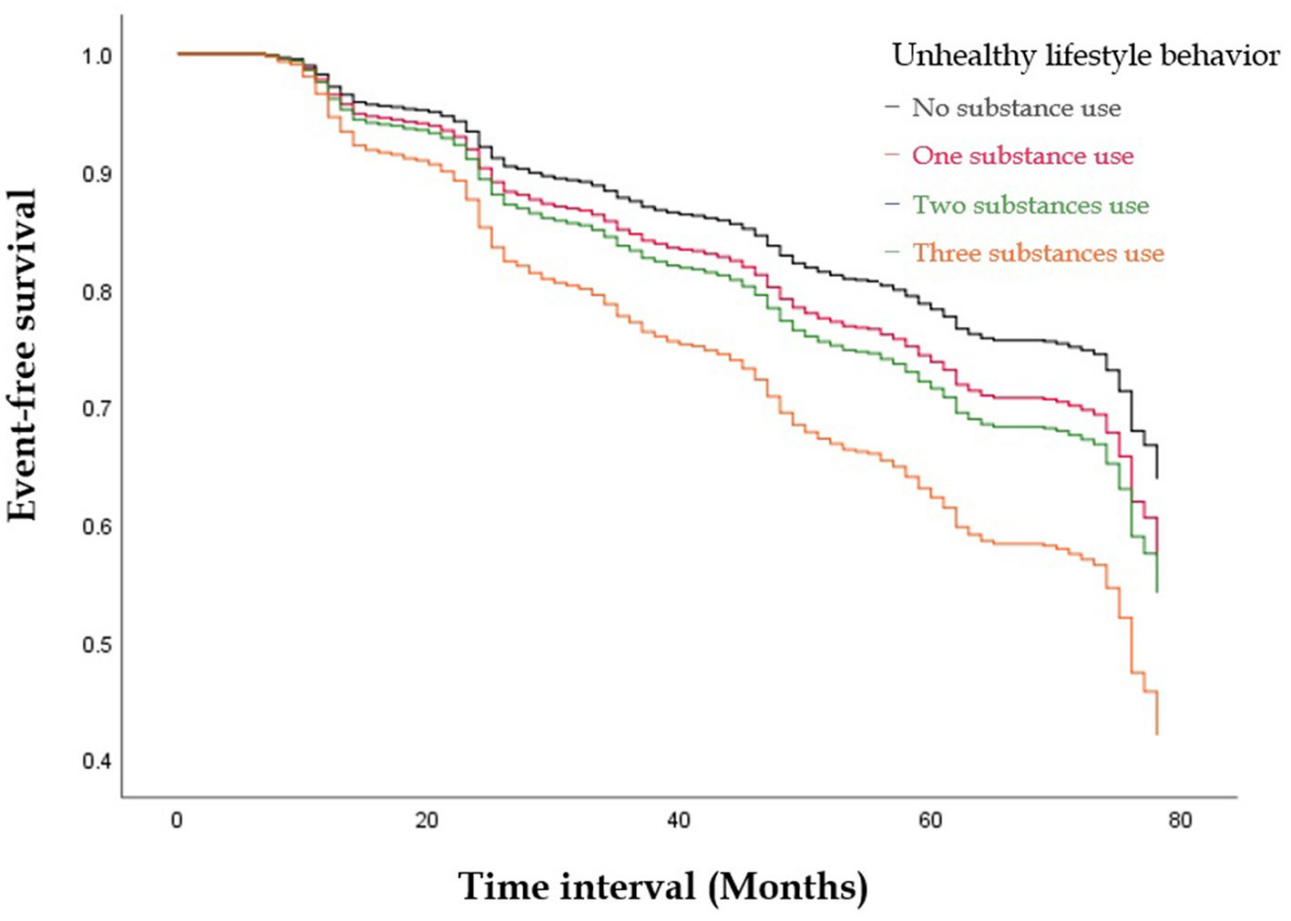
The Kaplan-Meier Curve illustrate the event-free survival curves for the combined substances use groups during the study period. The event-free survival probability decreased with an increasing number of substances used, with the lowest probability observed among participants who reported three substances use.

Figures 1, 2 illustrate the event-free survival curves for different substance use groups and the combined substance use groups during the study period. Those who reported only active cigarette smoking and those who reported only alcohol consumption had the lowest event-free survival probability, followed by those who reported only active betel nut chewing, as compared to those with no substance use (Figure 1). The event-free survival probability decreased with an increasing number of substances used, with the lowest probability observed among participants who reported three-substance use (Figure 2).

## Discussion

Our findings suggest that a combined use of alcohol, betel nuts, and cigarettes could significantly increase the risk of new-onset MetS in military young adults, with a clear dose-response relationship. Moreover, both cigarette smoking and alcohol intake were significantly associated with a 33% and 21% increased risk of new-onset MetS before midlife, respectively. Although betel nut use alone was not significantly associated with new-onset MetS, possibly due to a limited sample size with insufficient power for the analysis, it may still contribute to the increased risk of MetS when used in combination with other substances. Furthermore, the combined substance use was associated with a greater risk of incident MetS, particularly in those without baseline low HDL-C, indicating that HDL-C may be a vulnerable target that is easily affected by exposure to these toxic substances.

The association between each substance use and the risk of new-onset MetS is consistent with previous studies. Some meta-analyses have revealed a positive association between alcohol consumption and the risk of developing MetS (30, 31). Since alcohol intake was not allowed on military bases, most of the alcohol consumption in this study should be classified as mild, which emphasizes the impact of alcoholic beverages, even with mild consumption, on developing new-onset MetS in young adults. However, several cross-sectional studies have shown an inverse association between mild or moderate alcohol consumption and the prevalence of MetS and related comorbidities, such as diabetes and hypertension (13, 32). At baseline of this study, we also performed a logistic regression analysis of alcohol consumption alone for the prevalent MetS in participants aged 18–39 years, which revealed no association in the crude model and with adjustments for the covariates in model 1 [odds ratio (OR): 0.94 (0.69–1.26)] (Supplementary Table 1). The inconsistent findings may arise from the use of different methodologies; cohort studies can clarify the temporal association and provide a higher level of evidence compared to cross-sectional studies.

The association between cigarette smoking and metabolic syndrome (MetS) has been well-established in numerous studies (33–35). Our findings are consistent with the existing literature, as we observed a significant association between cigarette smoking and an increased risk of MetS, even after adjusting for potential confounders (HR 1.33, 95% CI 1.02–1.72 in Model 3). The mechanisms underlying this association are complex and multifaceted, involving various physiological pathways. Cigarette smoking has been shown to induce insulin resistance, a key component of MetS, through several mechanisms. Nicotine, a major constituent of cigarette smoke, was found to impair insulin sensitivity by activating the sympathetic nervous system and increasing lipolysis, leading to elevated free fatty acids and decreased glucose uptake by skeletal muscles (36). In addition, smoking is associated with increased pro-inflammatory cytokines, such as tumor necrosis factor-alpha (TNF-α) and interleukin-6 (IL-6), which can further contribute to insulin resistance (37). Furthermore, cigarette smoking has been linked to abdominal obesity, another critical component of MetS (35, 38). Smoking is also associated with an unfavorable lipid profile, characterized by lower levels of HDL-C and higher levels of triglycerides (39, 40), which are both components of MetS.

The mechanisms underlying the association between betel nut chewing and MetS are not fully understood but are thought to involve multiple pathways. Betel nut contains several active ingredients such as arecoline, which has been revealed the adverse effects on glucose and lipid metabolism. Animal studies have revealed that arecoline can induce insulin resistance by impairing insulin signaling and glucose uptake in skeletal muscle cells (41, 42). Arecoline can also stimulate the production of pro-inflammatory cytokines, such as TNF-α and IL-6, which are known to lead to insulin resistance and MetS (43, 44). In addition, Betel nut chewing has been associated with central obesity, a key component of MetS. A study by Lin et al. (9) found that betel nut chewers had significantly greater waist circumference and body fat percentage compared to non-chewers, even with adjustments for potential confounders. The mechanisms behind this association are not entirely understood but may involve the effects of betel nut on appetite regulation and energy balance (45). In this study, betel nut chewing was not significantly associated with incident MetS, probably due to a selection bias that more than half of only betel nut chewers having MetS at baseline were removed from the cohort for analysis. On the contrary, we found an association between betel nut chewing and prevalent MetS in the cross-sectional analysis (Supplementary Table 1).

To our knowledge, military personnel were predisposed to using substances. In the national statistics in Taiwan in 2014, the age- and sex-adjusted prevalence of cigarette smoking, alcohol intake and betel nut chewing was 26.3%, 26.2%, and 8.7%, respectively in the general population (46), which were less than that in our military cohort at baseline. However, military personnel were obliged to keep superior fitness that the mean waist circumference in our military cohort was 81 cm which was lower than the sex- and age- adjusted mean of the general population (88 cm) (47). Although previous studies have found an association between cigarette smoking, alcohol consumption, and betel nut chewing with a greater risk of MetS in the general population, the potential synergistic effect of these substances use have not been thoroughly investigated among military personnel. Our study expands upon the existing literature by examining the combined effect of these substances use on the incidence of MetS in a military cohort. We found that the risk of MetS increased with the number of substances use, with the greatest risk in those who reported using all substances (alcohol, tobacco, and betel nut), and the association was not affected by physical activity levels at baseline. This finding highlights the importance of considering the synergistic effect of multiple unhealthy lifestyle behaviors when assessing the risk of MetS and developing preventive strategies.

Interestingly, our subgroup analyses revealed that the associations of two and three substance use with MetS risk were more pronounced among participants without baseline low HDL-C levels. The mechanisms by which substance use overcomes the protective effects of high HDL-C on the risk of MetS are likely multifaceted and involve the interplay between various components of MetS. Cigarette smoking has been shown to reduce HDL-C levels, potentially leading to the increased incidence of MetS (48). Similarly, betel nut chewing has been associated with low HDL-C levels, and this effect may be more prominent than its influence on other MetS features, e.g. elevated TG and BP (7). For alcohol use, the association with reduced HDL-C was inconsistent. Several cross-sectional studies demonstrated that low or moderate alcohol use may increase HDL-C levels (49), whereas long-term alcohol use was linked to a higher possibility of low HDL-C in a Korean population study (50), suggesting that the relationship between alcohol use and HDL-C is complex and may depend on the duration and level of alcohol intake. Multiple substances use may have a synergic effect on the reduced HDL-C levels and metabolic health deterioration. For instance, the combination of active smoking and betel nut chewing may result in a more pronounced reduction in HDL-C level, and the detrimental effects of alcohol use on increasing serum triglyceride levels and central obesity (50) may further increase the incidence of MetS, even in the presence of initially normal HDL-C levels. As this is a complex issue, because each of substances can alter HDL-C levels independently and in different ways and directions, further studies have to be conducted to verify the synergic effects between the substances use on the changes in HDL-C levels.

The strengths of our study include the large sample size, the prospective cohort design, and the comprehensive assessment of lifestyle factors and metabolic abnormalities. However, some limitations should be acknowledged. First, the use of self-reported data on substance use may be subject to underreporting or recall bias. Second, since the study population consisted only of young military personnel in Taiwan who were characterized by being physically fit and having higher mental stress, our findings may have limited generalizability to other populations. Third, although we adjusted many baseline potential confounders, residual confounding cannot be entirely ruled out. For instance, the behavior of substances use may be merely a proxy of poor diet intake e.g., carbohydrates, which are usually accompanied with these substances use. Since there were no information on diet consumption, further study should be performed to clarify the confounding effect between habitual substances use and the risk of MetS. Finally, former smokers could not be differentiated from never smokers in our questionnaire which could not further analyze their association with new-onset MetS. Also, the overlapping periods of use for individuals who reported using two or more of alcohol, tobacco, and betel nuts were not available.

## Conclusion

Our study suggests that the combined use of alcohol, tobacco, and betel nut is associated with an increased risk of new-onset MetS in military personnel. The findings underscore the importance of adopting a comprehensive approach to preventive measures, targeting high-risk individuals who have simultaneous multiple substance use and potentially consume unhealthy diet. Future study should focus on developing and evaluating interventions that promote the cessation of these unhealthy behaviors, especially in military young adults, to reduce the risk of MetS and its associated health consequences.

## Data availability statement

The raw data supporting the conclusions of this article will be made available by the authors, without undue reservation.

## Ethics statement

The studies involving humans were approved by Institutional Review Board of the Mennonite Christian Hospital (certificate no. 16-05-008) in Taiwan. The studies were conducted in accordance with the local legislation and institutional requirements. The participants provided their written informed consent to participate in this study.

## Author contributions

W-NL: Conceptualization, Investigation, Methodology, Validation, Writing – original draft. Y-CH: Conceptualization, Data curation, Investigation, Visualization, Writing – original draft. Y-PL: Investigation, Supervision, Visualization, Writing – review & editing. K-ZT: Data curation, Formal analysis, Investigation, Methodology, Resources, Writing – review & editing. Y-CC: Investigation, Supervision, Validation, Visualization, Writing – review & editing. P-YL: Investigation, Supervision, Validation, Visualization, Writing – review & editing. G-ML: Conceptualization, Data curation, Funding acquisition, Investigation, Methodology, Project administration, Resources, Supervision, Validation, Visualization, Writing – review & editing.
