# Supplementary material for: Substance use and incidence of metabolic syndrome before midlife among military adults: the CHIEF cohort study
Source: Front Public Health. 2024 Jun 4;12:1406524. doi: 10.3389/fpubh.2024.1406524 (PMC11184061; doi:10.3389/fpubh.2024.1406524)
Supplement: Supplementary file 1 [file Table_1.docx]

Supplemental Table 1. Multiple Logistic Regression Analysis for Incidence of Metabolic Syndrome with Unhealthy Lifestyle Behaviors (N =3,044)

|  |  |  |  | Model 1 |  |  |  | Model 2 |  |
| --- | --- | --- | --- | --- | --- | --- | --- | --- | --- |
|  | N | MetS | OR | 95% CI | p |  | OR | 95% CI | p |
| No substance use | 1880 | 165 | 1.00 |  |  |  | 1.00 |  |  |
| Only alcohol drinking | 699 | 73 | 1.21 | 0.91 – 1.62 | 0.19 |  | 0.94 | 0.69 – 1.26 | 0.66 |
| Only betel nut chewing | 18 | 5 | 4.00 | 1.41 – 11.35 | 0.009 |  | 3.33 | 1.13 – 9.78 | 0.02 |
| Only cigarette smoking | 447 | 54 | 1.43 | 1.03 – 1.98 | 0.03 |  | 1.31 | 0.94 – 1.83 | 0.11 |

Data are presented as hazard ratio (HR) and 95% confidence interval (CI).

Multivariable Model 1 was adjusted with age, sex and physical activity level.

Multivariable Model 2 was adjusted with age, sex, physical activity level and body mass index.

Supplemental Table 2. Multiple Logistic Regression Analysis for Incidence of Metabolic Syndrome with Unhealthy Lifestyle Behaviors (N =4,080)

|  |  | Model 1 |  |  |  | Model 2 |  |
| --- | --- | --- | --- | --- | --- | --- | --- |
|  | OR | 95% CI | p |  | OR | 95% CI | p |
| No substance use | 1.000 |  |  |  | 1.000 |  |  |
| One substance use | 1.329 | 1.044 – 1.693 | 0.02 |  | 1.102 | 0.859 – 1.414 | 0.44 |
| Two substances use | 1.539 | 1.175 – 2.017 | 0.002 |  | 1.266 | 0.959 – 1.671 | 0.09 |
| Three substances use | 2.808 | 2.052 – 3.842 | <0.001 |  | 2.432 | 1.760 – 3.362 | <0.001 |

With, age, sex and PA level adjustment
